# Health knowledge, attitudes and practices of family planning service providers and clients in Akwapim North District of Ghana

**DOI:** 10.1186/s40834-016-0016-3

**Published:** 2016-03-07

**Authors:** Margaret Duah Atuahene, Esther Oku Afari, Martin Adjuik, Samuel Obed

**Affiliations:** 1 School of Public Health, University of Ghana, Accra, Ghana; 2 Ministry of Health, Akwapim, Ghana; 3 INDEPTH Network Secretariat, Accra, Ghana; 4 Korle-Bu Teaching Hospital, Accra, Ghana

**Keywords:** Reproductive Health, Family Planning, Sub-Saharan Africa, Provider, Contraceptive, Client, Survey, Service provision

## Abstract

**Background:**

Family planning services help save lives by reducing women’s exposure to risks of child birth and abortion. While family planning services provide measures to prevent unintended pregnancies and time the formation of families, the acceptability and coverage is still very low worldwide. Some of the reasons for this include poor quality of service, unavailability of range of methods, fear of opposition from partners, side effects and health concerns among others.

About 40 % of the world’s 215,000 annual deaths in childbirth occur in the Sub-Saharan region. In Ghana, urban–rural fertility differences range from two to three children. The acceptability and coverage of family planning are still low and in the study area in particular.

**Methods:**

We sought to examine factors that contribute to low acceptability and coverage of family planning services in a sub-urban community with a design of quantitative cross-sectional. Ethical approval was given by the Ghana Health Service. Midwives and community health nurses who provide family planning services were interviewed. Exit-interview was also conducted with women receiving a variety of outpatient services.

**Results:**

Most of the women in this study (48.7 %) were in the 25–34 age range and were either married (42.8 %) or cohabiting (40.5 %). Majority of these women (67.7 %) have middle/Junior high level of formal education with a modal parity of two. Sixty eight (68) clients were identified as current family planning users. About 6.0 % and 4.5 % were dissatisfied about auditory and visual privacy during counselling respectively. This was confirmed by providers who attributed it to inappropriate facility layout. Most of the clients (79.1 %) were not given educational materials although 88.8 % were talked to about family planning and this could be due to unavailability of these hand-outs.

Though clients show satisfaction of services received, providers did not follow standard protocols with as much as 73.7 % faced with challenges in provision of services which were attributed to improper facility layout and lack of furniture. About 77.2 % were willing to provide short term methods, while 91.2 % wanted to provide long term methods. As much as 93.3 % of the women said they would have liked providers give more detailed information on family planning. While most of the women (88.3 %) used injectables, only 6.1 % and 0.9 % used Implants and IUD respectively.

**Conclusions:**

Finding ways to improve client privacy through good facility layout will ensure visual and auditory privacy to enhance family planning service provision and uptake. Continuous competency training will assist providers design innovative action plans and meet client satisfaction needs.

## Background

### Unintended pregnancy

About 80 million unintended pregnancies are estimated to occur worldwide annually and in developing countries more than one-third of all pregnancies are considered unintended and about 19 % will end up in abortion, which are most often unsafe accounting for 13 % of all maternal deaths globally [1, 2]. Two-thirds of unintended pregnancies in developing countries occur among women who are not using any method of contraception. This indicates the failure to take necessary decisions to prevent and avoid unwanted pregnancies [3].

Globally, 50 million women resort to induced abortion which ultimately results in high maternal morbidity and mortality. In 2007, an estimated 536,000 maternal deaths occurred globally with 1 % in developed countries and 50 % in Sub-Saharan Africa [4].

### Contraceptive uptake

Contraceptive uptake is low in rural areas and lag behind urban populations [5]. In Ghana, urban–rural fertility differences range from two to three [6]. The poorest rural wealth quintiles have modern contraceptive levels ranging from less than 5 % to about 15 % of eligible women [7]. Current evidence shows slow progress in expanding the use of contraceptives by women of low socioeconomic status [8].

Access to contraceptives can also be inhibited by certain provider practices such as use of excessively restrictive medical criteria or provider bias against certain methods; these practices are often referred to as medical barriers. Therefore, identifying and addressing these barriers may facilitate improvements in quality of care and subsequently uptake of family planning services. While quality of family planning services may be good, clients report barriers such as misinformation and provider disrespect [9]. In other words, lack of education materials or being misinformed, and showing no dignity for clients were considered as barriers to accessing family planning and other health delivery services.

### Benefits of family planning

Family planning can reduce the number of deaths among women by reducing the number of women who are at risk by averting unintended pregnancy, which accounts for about 30 % of all births in Sub-Saharan Africa [10]. Therefore, family planning can reduce maternal mortality though access to quality of service is a major concern [11]. Other benefits of family planning include prevention of cancers and sexually transmitted diseases [12].

The benefits are well established [13, 14]. In some African countries, about one-third of women’s need for family planning is unmet, such as Rwanda with 38 % and Uganda with 41 %. Encouragingly, an analysis of survey data showed a significant level of women with unmet need who had never used family planning intended to do so [15].

### Birth spacing

The growing use of contraception around the world has given couples the ability to choose the number and spacing of their children which have tremendous lifesaving benefits. Yet despite these impressive gains, contraceptive use is still low and the need for contraception high in some of the world poorest and most populous places [16]. In the past 20 years alone, maternal deaths in developing countries have been reduced by 40 % [17]. Family planning programmes have been effective in the past and have played a major part in increasing the use of contraception from 10 to 60 % and reducing average fertility in developing countries from 6 to 3 births per woman [18].

### Unmet needs

Fertility rates in sub-Saharan Africa remain high and more than twice the global average [19–21]. Women who prefer spacing births but are not using contraception are considered to have unmet need [22]. While access also to affordable modern methods of contraception is sometimes a problem, many women reported other reasons for not using family planning, such as not perceiving themselves at risk of pregnancy because they did not have sex frequently, were going through menopause or were lactating. But these situations do not offer protection against pregnancy in all cases [23].

### Accessibility

Women living in urban areas may have greater access to a wide variety of contraceptive services and methods as compared to rural women [24]. In this light, it is obvious that the poor cannot access family planning counselling and other services, due to high cost and proximity accounting for unmet needs and low family planning coverage.

Facilitating access to modern contraceptives for women with unmet needs has the potential benefit of improving maternal child health and reducing mortalities [12, 25–28].

This study was to assess knowledge, attitude, perceptions and practices of providers, whilst attempting to understand the perspectives, needs and motivations of clients that may help address barriers to uptake and adherence to family planning standard and procedures.

## Methods

A cross-sectional quantitative descriptive study was designed to collect information on knowledge, attitude and practices in provision of family planning services among women of reproductive age and service providers in the Akwapim North District. The district has an estimated population of 122,063 and 3.1 % growth rate. There are two hospitals, seven health centres and sixteen Community Health Planning Services (CHPS) compounds. There is an estimated 28,075 women in the fertility age, with family planning coverage of roughly 27 %.

### Sampling of study participants

The sample size for the study was computed as follows: The population proportion formula: n = z^2^*p*(1-p)/d^2^, was applied, where n is the desired sample size, z is the standard normal deviate at the 95 % confidence level, p is the proportion of women who assessed family planning services in the study area and d is degree of accuracy desired; and. Thus using z = 1.96, *p* = 0.27, and d = 0.05, the calculated sample size for clients (women in reproductive age) was 303.

From the district records, 7,580 clients were active family planning users in 2012. Therefore, applying the finite population correction factor, where n1 is the required sample size; and N is the population of clients who utilizes family planning services; a final sample size of 291 was estimated. The study population comprised women of reproductive age (15–49 years) who sought family planning services regardless of their reproductive status and they have to be residents in the district.

### Study population

The study population for this research is defined as women of reproductive age (15–49 years) resident in the district who receive family planning services and providers who offer family planning services made up of mostly midwives and nurses. They included current family planning users and other women seeking ANC, post-partum and STI services. Also forming part of the study population were midwives and community health nurses who are providers of family planning services. Family planning services are accessed in these comprehensive clinics that provide family planning as part of inclusive health delivery. As a result of cultural stigmatization associated with family planning accessibility, most women prefer seeking services in clinics where FP services are provided alongside with other services. This is deliberately arranged to prevent being stigmatized for only family planning. In other words, it is shrouded in secrecy. In this study sixty eight (68) clients were identified as current family planning users as indicated in Tables 2 and 3.

All midwives and community health nurses in the study area were interviewed for information on access and utilization of family planning services among women of reproductive age. Exit-interviews were conducted with women attending family planning clinic and ante-natal care (ANC). A combined checklist and structured questionnaire were used to collect information from service providers while structured questionnaire were administered to clients. Clients responded in English and local dialects for those who required translation. Information was kept confidential and names of participants were not included in the analysis or reported in the study results.

### Quality control

Data were checked for completeness and accuracy. Regular verification and validation of data were done with all inconsistencies being checked and resolved with the research assistants and data entry clerks. The data were entered using EPIDATA 3.1 software and imported to statistical software, STATA version 11.

To ensure quality and validity of data, the following measures were put in place. Research assistants with requisite background were recruited and trained on data collection guidelines. The survey was administered by the authors and assisted by trained research assistants. Collected data were checked to ensure that all questions were answered. Probing was done where necessary during interviewing.

Errors detected were discussed and necessary corrections made. Every questionnaire was marked to prevent double entry. Financial constraints - funds were not readily available. Access to some parts of study area was very difficult due to the poor nature roads. Some measures were taken which included researchers being objective in dealing with respondents, financing of the study was from researchers’ own resources and managed to get to catchments areas by their own means of transport.

### Ethical review

Ethical clearance was obtained from the Ghana Health Service’s Ethical Review Committee and permission was sought from the District Health Administration. All participants were assured of anonymity and confidentiality and participation was voluntary. They were assured that the results will purely be used for research purposes.

## Results

### Background characteristics of family planning clients

The majority of family planning clients in the exit-interview (48.7 %) were aged 25–34 years, 30.1 % were aged 15–24 years, and 19.0 % were aged 35–44 years (Table 1). Out of the total (269), 42.8 % were married and 40.5 % cohabited. The remaining 16.7 % were either single, or never married. 67.7 % of the women had Middle/Junior High level of education and 24.9 % and 20.8 % had parity of two and four respectively. The modal number of children alive was two children (26.0 %) followed by one child (21.9 %).

**Table 1:** Basic Demographic characteristics of Family Planning Clients (*N* = 269)

Variables	Number (n)	Proportion (%)
Age Group		
15–24	81	30.1
25–34	131	48.7
35–44	51	19.0
45+	6	2.2
Marital status		
Married	115	42.8
Cohabiting	109	40.5
Single, Never married	45	16.7
Level of Education		
None	4	1.5
Primary	50	18.6
Middle/Junior High	182	67.7
Secondary	33	12.3
Parity		
Parity 0	43	16.0
Parity 1	55	20.5
Parity 2	67	24.9
Parity 3	48	17.8
Parity 4	56	20.8
Number of children alive		
None	30	11.2
One	59	21.9
Two	70	26.0
Three	51	19.0
Four	37	13.8
Five	22	8.2

Source: Fieldwork, June 2014

### Current family planning clients assessment of provider knowledge and skills (*N* = 68)

Table 2 shows the results of assessment of knowledge and skills of family planning services of providers from the clients’ perspectives. Clients were asked about the desire to have children before the introduction of family planning service. Women who desired children in the future accounted for 75.1 % (51), a sizable percentage while 23.0 % indicated no desire for future children. In terms of hand-outs for FP materials, (IEC) 79.2 % said they were not provided with them. As much as 93.3 % said they would have liked to receive hand-outs. About 88.9 % accounted for those who were talked to about family planning. On the discussion of new methods by providers, 50 % of clients did not indicate affirmation. Not all participants were existing family planning service users. Some of the women were ante-natal patients and may not have been exposed to family planning before their visits to the clinics. Still, some could have been first time mothers, with unplanned pregnancies who may not have used family planning at all in their lives but desired to do so shortly or in the future. All participants were exposed to family planning information before they exited the clinics or health centres. While most (88.2 %) used injectables, only 6.1 % and 0.9 % used Implants and IUD, respectively. A large proportion (98.3 %) said the waiting time was reasonable and 94.4 % said they would recommend the facility to a friend for family planning services.

**Table 2:** Current Family Planning Clients’ Assessment of Provider Knowledge and Skills (*N* = 68)

Variables	Number(n)	Proportion (%)
Asked about children in the future		
Yes	51	75.1
No	16	23
Don’t know	1	1.9
Received FP Material		
Yes	14	20.8
No	54	79.2
Talked to about FP		
Yes	60	88.9
No	8	11.1
Would have liked hand-outs about FP		
Yes	63	93.3
No	5	6.7
Method using currently		
Oral pills	3	4.8
Injectable	60	88.2
Implants	4	6.1
IUD	1	0.9
Provider discussed new method		
Yes	18	26.4
No	50	73.6
Waiting time for FP		
Too long	1	1.7
Reasonable	67	98.3
You will recommend the facility to a friend		
Yes	64	94.4
No	4	5.6

Source: Fieldwork, June 2014

### Level of satisfaction of FP services by current FP clients

All current family planning clients said they were satisfied with discussions on problems and concerns, explanation given, quality of examination, visual and auditory privacy, cleanliness and interaction with facility staff as indicated in Table 3.

**Table 3:** Level of satisfaction of FP services by current FP clients

Areas of satisfaction of services	Level of satisfaction of FP services		
	Satisfied	Dissatisfied	Undecided
Discussion of concerns with provider	68 (100)	0 (0)	0 (0)
Explanation provided about problem	68 (100)	0 (0)	0 (0)
Quality of examination provided by provider	62 (91.8)	5 (7.4)	1 (0.8)
Visual privacy during examination	65 (95.2)	3 (4.8)	0 (0)
Auditory privacy during discussion	64 (93.7)	4 (6.3)	0 (0)
Cleanliness of the facility	68 (100)	0 (0)	0 (0)
Treatment received from facility staff	68 (100)	0 (0)	0 (0)

Source: Fieldwork, June 2014

### Characteristics of FP facilities and service providers (*N* = 70)

Table 4 shows the basic characteristics of family planning facilities and service providers. The number of FP providers interviewed was 70. A proportion of 82.1 % were females, 78.9 % constituted Community Health Nurses (CHN) and 49.1 % had been in their current position for up to 2 years. Their mean age was 31.5 years with a standard deviation of 9.8 years.

**Table 4:** Characteristics of FP facilities and service providers (*N* = 70)

Characteristics	Number(n)	Proportion (%)
Type of facility		
Hospital	5	7.1
Health Centre	25	35.7
CHPS Compound	30	42.9
Others	10	14.3
Gender of service provider		
Male	13	17.9
Female	57	82.1
Professional qualification		
Midwife	7	10.5
SRN	1	1.8
CHN	55	78.9
Enrolled Nurse	3	3.5
HCA	1	1.8
Others	3	3.5
Length of service		
<1 year	15	21.1
1–2 years	34	49.1
3 + years	21	29.8
Age in years (mean ± SD)	31.5 ± 9.8	

Source: Fieldwork, June 2014

### FP service provision from provider perspective

Table 5 indicates family planning service provision from the perspective of the provider. Most (75.4 %) said they receive their supplies monthly. About 77.2 % provided information, counselling, short term method and with 91.2 % interested in long term methods. As much as 73.7 % indicated facing challenges including improper facility layout and lack of furniture.

**Table 5:** FP Service Provision from Provider Perspective

Variables	Number (n)	Proportion (%)
Frequency of FP supply		
Weekly	1	1.8
Monthly	53	75.4
Quarterly	15	21.1
Others	1	1.8
FP Services willing to Provide FP information		
Yes	54	77.2
No	16	22.8
FP Counselling		
Yes	54	77.2
No	16	22.8
FP Short Term methods		
Yes	54	77.2
No	16	22.8
FP Long term methods		
Yes	64	91.2
No	6	8.8
Faced with challenges in FP services provision		
Yes	52	73.7
No	18	26.3

Source: Fieldwork, June 2014

## Discussions

### Socio-demographic profile

Most of the women (48.7 %) aged 25–34 and were either married (42.8 %) or cohabiting (40.5 %). This means that most of the women are in active reproductive age and in active nuptial union. Majority of these women also have more middle/Junior high level of schooling with a parity of two. This revealed that, those who had received some level of formal education are in the majority, compared to those without any form of education. Studies have shown that, further educational and class differences between clients and providers often limit clients’ ability to access services [29].

### Confidentiality and privacy

The desire to have more children supports a study done in the region of Tigray which showed that African men generally, desire more children because of social and economic benefits. Studies have shown that clients, particularly those who obtain services in secret, report higher satisfaction with providers who keep their needs and personal information confidential [30]. Therefore, lack of privacy can violate women’s sense of modesty and make it more difficult to participate actively in family planning services.

Though clients generally indicate satisfaction about counselling, quality of examination, cleanliness of facility and treatment, some 6.0 % and 4.5 % were dissatisfied about auditory and visual privacy respectively. In a similar study in Zambia, clients based their judgment on how thoroughly they were examined due to confidentiality and privacy [31]. In Chile, all but one respondent mentioned the facility’s cleanliness as a sign of the quality of the clinic’s services [32].

### Cultural perspective and family planning services

It was also revealed [33] that in a few places, obtaining and using contraceptives can be a difficult and risky decision that can lead to abandonment, violence, ostracism, or divorce hence women need assurance of absolute confidentiality. This result is similar to a recent research in which clients’ perspectives on services they receive are essential part of understanding and assessing quality of care [34]. Other studies have shown that, clients’ perceptions are shaped by their cultural values, previous experiences, perceptions of the role of the health system and interactions with providers. Their perceptions affect how clients view the risks and benefits of care [35].

Again, studies have shown that clients may also indicate that they are satisfied with care because they want to please the interviewer, worry that care may be withheld in the future, or have some cultural or other reason to fear complaining [36]. Many clients have limited options and have never experienced any other standards of care.

### Provider and client relationship

The results show that, the providers were able to build a good rapport with their clients by greeting and introducing themselves. This finding contradicts that in a study by [9] where providers failed to greet clients in a respectful or friendly manner and generally displayed negative attitudes towards clients. In many societies, courtesy is a sign that the client is regarded as the provider’s equal. Research shows that the provider’s tone, manner, and modes of speech are important to clients [37–39].

Studies found that women are more likely to seek out and continue using family planning services if they receive respectful and friendly treatment [11, 32, 40], as cited in [33, 41]. A good proportion of the clients (38.6 % and 32.9 %) were not asked about previous pregnancy/reproductive and family planning history respectively.

### Information and educational materials

Results from the checklist shows that greater proportions (61.4 %) of them were not provided with educational hand-outs. This confirms the result from the client exit interview where most (79.2 %) of the clients said they were not given family planning materials. Some 22.8 % were not willing to provide FP counselling and short term methods though most (88.3 %) of the clients are on injectables (short-term). About 26.3 % of the providers said they face challenges in the provision of services and majority (91.2 %) prescribed long term methods. Research has shown that providing more complete and accurate counselling that is tailored to the client’s needs, including providing hand-outs has been associated with higher levels of client satisfaction, as well as higher contraceptive prevalence and client retention [36].

### Limitation of the study

This study is not without limitations. The sensitive nature of some questions may have resulted in some bias. Thus there could be the possibility of reporting behaviours that are socially desirable. Subjectivity which could have resulted in bias, in an effort to relegate our values and beliefs to the background, when dealing with respondents. However, the purpose of the study was explained to the best of our ability. This was done to prepare participants to provide objective views to help improve family planning services in the community.

## Conclusions and recommendations

Although family planning clients show a general satisfaction about the provision of family planning services, it was clear that providers were not following the standard or protocols when attending to clients. These depend on the counselling skills, adherence to principles in phases and steps during encounter with clients.

Family planning clients in study area do not identify any negative attitude of service providers that hinder access to family planning services but were concerned about their visual and auditory privacy. This was again confirmed by service providers who said the facility layout is a challenge in service provision. Identifying the problems, retraining providers, and using clear protocols for engaging clients resulted in much improved client-provider interactions and addressed the needs of clients, providers, and program managers.

Finding ways to enhance client privacy through good facility layout for visual and auditory privacy, increase provider skills and expertise to ensure all phases and steps in family planning counselling (rapport building, exploration, decision making and implementing the decision) are followed. This could be done through good supervision and refresher courses. Training is especially needed to equip providers offer short and long term services to enhance and scale up family planning services in communities.

## Abbreviations

WHO: World Health Organization
UNFPA: United Nation’s Fund for Population Activities
CYP: Couple year protection
FP: Family planning
CHPS: Community Health Planning Services
TF: Total fertility rate
CHN: Community health nurses SRN, senior registered nurse
SRN: senior registered nurse
HCA: Health Care Administrator
IEC: Information, education, and communication
